# Editorial: Population and comparative genomics of plant pathogenic bacteria

**DOI:** 10.3389/fmicb.2022.1012034

**Published:** 2022-08-31

**Authors:** Sujan Timilsina, Erica M. Goss, Ralf Koebnik, Neha Potnis, Jeffrey B. Jones

**Affiliations:** ^1^Department of Plant Pathology, University of Florida, Gainesville, FL, United States; ^2^Emerging Pathogens Institute, University of Florida, Gainesville, FL, United States; ^3^Plant Health Institute of Montpellier, University of Montpellier, CIRAD, INRAE, Institut Agro, IRD, Montpellier, France; ^4^Department of Entomology and Plant Pathology, Auburn University, Auburn, AL, United States

**Keywords:** taxonomy, evolution, ecology, effector, host specificity

Plant pathogenic bacteria are among the major challenges in crop production, amplified by their continuous and rapid evolution and resulting in the emergence of virulent, competitive, and elusive plant pathogens. To further understand these bacteria, their interactions with host plants, and their responses to disease management, comprehensive methods are necessary. In recent years, phytobacteriology research has used population and comparative studies to quantify the diversity of plant pathogenic bacteria and their virulence mechanisms, including the effectors secreted from different protein secretion systems, phytohormones, or toxins that mediate their interactions with the hosts and/or competing microbes in the phyllosphere. Studies comparing strains over time, within and among geographic regions, and across phylogenetic trees has provided insights into scales of variation and changes in pathogens over time and in response to selection pressures. In this series of articles, population and comparative genomics were used to investigate plant pathogens representing diverse bacterial genera including Gram-negative bacteria composed of facultative anaerobes (i.e.*, Dickeya, Pantoea*, and *Pectobacterium*) as well as obligate aerobes (i.e., *Pseudomonas, Xanthomonas*, and *Xylella*) as well as the Gram-positive genus, *Clavibacter*.

## Genomics approaches clarify taxonomy and provide resources for comparative studies

Since ANI has been introduced as a means to reevaluate taxonomic assignments, genomics approaches have been used for comparative studies at different taxonomic levels. In this Focus Issue, reference pathotype strains of all described *Xanthomonas translucens* pathovars were sequenced using PacBio long-read technology and complete genome sequences were assembled, which gave robust proof for three major clades in this species, each with distinct host preferences (Goettelmann et al.). Moreover, type III effector (T3E) profiling revealed 21 TAL effector classes and 29 additional type III effectors, some of them displaying clade or strain specificity. Further investigation of these genes could help to identify genes that are critically involved in pathogenicity and/or host adaptation, setting the grounds for the development of new resistant cultivars.

Expansion of whole genome sequencing of species within the same genus has offered insighst into host specialization and into phylogenetic relationships within and between species within genera. Prior to the identification of the *Xylella* species, *X. taiwanensis*, characterization was restricted to different subspecies of *Xylella fastidiosa*, but lacked analyses at the genus level due to the lack of genome sequences for multiple species (Weng et al.). As a result of having the *X. taiwanensis* genome available as an outgroup in phylogenetic comparisons of *X. fastidiosa* subspecies, there was strong support for *X. fastidiosa* subspecies *pauca* being the basal lineage of *X. fastidiosa* and for *Xylella* being derived from the paraphyletic genus *Xanthomonas*.

Genome sequencing is also revealing previously unrecognized diversity, as is illustrated by the characterization of *Xanthomonas* causing bacterial leaf canker on water spinach (*Ipomoea aquatic*) by Hu et al. a survey of the disease in Dongguan City, China, where water spinach is grown in small plots, found the *Xanthomonas* causal agent along with *Pantoea*, which had a synergistic effect on disease. Whole genome sequencing using short and long-read technologies of a representative *Xanthomonas* strain showed >97% ANI to *X. perforans*, which is a classic pathogen of tomato, but was more recently also identified on pepper and eucalyptus. The ANI below 98% indicates that this is an atypical *X. perforans*. The catalog of effectors in this strain will advance efforts to understand the relationships between effector repertoires and host range in *Xanthomonas*.

Soft rot pectobacteria are devastating plant pathogens with a global distribution and a broad host range. To close the shortage for genome data of *Pectobacterium aroidearum*, the causal agent of soft rot on the popular houseplant *Syngonium podophyllum*. Xu et al. present the complete genome sequence of strain L6. About 10% of the predicted genes were potentially related to pathogenesis using the Virulence Factors of Pathogenic Bacteria database, including genes related to toxins, plant cell-wall degrading enzymes, and bacterial secretion systems. This study provides novel information for the discovery of potential pathogenicity factors and the development of more effective strategies against this pathogen.

Historically, less work has been done on Gram-positive plant pathogens. Among them, *Clavibacter michiganensis*, a member of the Actinobacteria, is a causal agent of bacterial canker of tomato. Oh et al. provide the complete genome sequence of the type strain of *C. michiganensis*. When compared with other strains, it turned out that the chromosomal DNA sequences were almost identical, whereas its plasmids were found to carry distinct gene content among *C. michiganensis* strains. The genome information of the type strain LMG 7333^T^ will help in understanding the genetic diversity of *Clavibacter*'s plasmid complements and how they may relate to virulence on host plants.

## Using genomics to investigate variation and evolution of type III effector repertoires

Traditionally, T3Es have been thought to be important host-range determinants. Such effectors have been characterized as avirulence factors that define race structure in plant pathogens. Understanding the race structure in pathogen population and factors associated with race specificity are of importance when assessing efficacy and durability of host resistance. Rosenthal et al. used a comparative genomics approach on genome sequences of different races of *Xanthomonas hortorum* pv. *vitians* and identified two T3Es, XopAQ and XopAF2, as potential mediators of gene-for-gene interactions between race 1 and 3 strains and wild lettuce. The close examination of the neighborhood regions of these effectors indicated proximity of *xopAF2* to prophage sequences, thus, likelihood of gain or loss of this effector in the pathogen population *via* phage-mediated transfer, in response to host selection pressure.

Shah et al. analyzed T3Es of *X. translucens* strains that cause bacterial leaf streak in small grain cereals, which include three pathovars: *cerealis, translucens*, and *undulosa*. Four effectors, XopAJ, XopAL1, XopE3, and XopM were found in all analyzed *X. translucens* pv. *translucens* strains, but not in members of the other two pathovars, *cerealis* and *undulosa*. Whether these effectors act as host specificity factors, as hypothesized by the authors, remains to be experimentally proven. Interesting differences were also reported in the TAL effectors, which activate the expression of host genes, including two novel dipeptides (called RVDs for Repeat Variable Diresidues) that contact the bound DNA and provide sequence specificity to these transcription activators (Shah et al.).

The *Pseudomonas syringae* species complex represents a classic system to address convergence of diverse strains belonging to distantly related phylogroups onto a common host. Ruinelli et al. conducted association analyses on genomes of *P. syringae* strains associated with diseases on *Prunus* as well as those from other hosts and from non-agricultural environments. They identified horizontal gene transfer (HGT) as a predominant evolutionary force that has mediated gain/loss of genes such as the T3E, *hopAY*, among strains belonging to diverse phylogroups in a relatively short evolutionary timescale. Such independent acquisition events of genes important for successful pathogen colonization, but also loss of specific genes or their inactivation, have shaped what we define as host ranges in plant pathogenic bacteria.

Heritable changes in bacteria, including effectors, often occur during conjugation or horizontal gene transfer of mobile genetic elements. Integrative Conjugative Elements (ICEs) are among the mobile replicons that combine the characteristics of plasmids with an ability to carry genes. ICEs are also known to carry cargo regions that can drastically change bacterial phenotypes, including their interactions with hosts. Baltrus et al. demonstrated that a T3E ICE from *P. syringae* pv. *maculicola*—PmaICE-DQ—can transfer to other strains *via* conjugation, resulting in new phenotypes of the recipient. Moreover, another ICE carrying multiple effector genes—PmaICE-AOAB—was found to be adjacent to the PmaICE-DQ. The study demonstrated the transfer of ICEs regions to naive strains. The presence of effectors within ICEs enables quick loss and gain of effectors, thus providing additional flexibility to the pathogen under selection pressure.

Whole genome sequencing can capture shifts in effector content in pathogens over time. Bernal et al. screened hundreds of *X. perforans* strains from tomato fields in the Midwestern USA over a period of 4 years, representing multiple varieties, seed producers, and growers. They found that the *X. perforans* population shifted from race T3 to race T4, mostly caused by a SNP in *avrXv3* that inactivated this effector gene by an early stop codon. While host selection is a likely explanation for the loss of effector function, few tomato varieties with resistance to *X. perforans* T3 made up <7% of the acreage in this geographic region. Analysis of whole genome sequences showed that most strains were nearly identical to each other. The authors discuss the possible mechanisms of persistence of this clonal population from year to year, which is critical knowledge needed to manage bacterial spot of tomato.

## Beyond type III effectors

A recent paradigm to explain genetic basis of host adaptation or specificity has suggested involvement of multiple genetic determinants (not limited to T3Es), each contributing small or large effects toward adaptation of pathogen to a specific host or tissue type. Liyanapathiranage et al. conducted large-scale comparative and evolutionary analyses to test the hypothesis that specific gain or loss events of multiple type VI secretion systems are not random, but occurred as independent events during the adaptation of *Xanthomonas* species to specific hosts. They observed that specific cases of loss or gain events happened in certain clades. Whether these secretion systems and their effectors contribute to an adaptation to vascular or non-vascular lifestyles or provide preference toward dicots or monocots remains to be addressed experimentally.

Exhaustive genomics resources stimulate comparative studies aiming at cataloging known and discovering new traits, thus better understanding how they are linked to the ecology of the pathogen. Bacterial toxin-antitoxin (TA) systems, consisting of two or more adjacent genes, are one of these traits of interest, which are implicated in genome maintenance, antibiotics persistence, phage defense, and virulence. Kandel et al. used bioinformatics tools to screen the genomes of hundreds of *P. syringae* strains representing the genetic and lifestyle diversity of the *P. syringae* species complex for TA systems. They show that *P. syringae* strains encode on average 15 TA systems per genome, which belong to 26 different families and are thought to target diverse cellular functions. Further functional characterization of the predicted TA systems could reveal how these widely prevalent gene modules may affect *P. syringae* ecology, virulence, and disease management practices.

Another approach to identify previously unknown virulence factors is using genome-wide association studies (GWAS). Agarwal et al. applied a GWAS approach on *Pantoea ananatis* that causes center rot of onion. This bacterium does not utilize the type II or type III secretion system, but possesses a biosynthetic gene cluster—HiVir/PASVIL—for production of a phosphonate secondary metabolite and also another gene cluster—*alt*—that confers tolerance to thiosulfinates. The pangenome association analyses indicated that although HGT events, including transfer of PASVIL cluster, may have contributed toward diversification and niche adaptation of *P. ananatis*, other factors (e.g. tyrosine kinase, N-acetylmuramoyl-L-alanine amidase, and HTH-type transcriptional regulator) are likely also involved in the pathogenicity of *P. ananatis* on onion.

High-throughput sequencing offers incredible opportunities for identifying genes involved in various microbial processes including fitness, virulence, and antibiotic resistance. Transposon mutagenesis was useful in identifying essential genes for growth on media as well as in planta. Random barcode transposon-site sequencing (RB-TnSeq) was used in comparing three *Dickeya* strains from two species to identify genes involved in fitness as measured by bacterial growth in potato tubers and growth in minimal and complex media (Helmann et al.). Notably, many of the metabolic traits that were required for growth in minimal medium were also important for efficient growth in potato tubers, such as amino acid, carbohydrate, and nucleotide biosynthesis. Moreover, growth of all three *Dickeya* species required the pectate degradation gene, *kduD*, whereas disruption in three putative DNA-binding proteins had different effects at the strain level.

The last two decades have witnessed adoption of comparative and population genomics methods being integrated into addressing several questions related to pathogen biology, epidemiology as well as translational research to guide disease management practices based on changes in pathogen population structure. The articles published in this Research Focus show such translational aspects, informing meaningful hypotheses that demand further functional genomics studies. Thanks to the plant pathologists who have taken initiative to sequence several hundreds, if not thousands, of genomes of their favorite bacterial genus, we have rich databases with genotypic and phenotypic data. The articles published in this issue also address this important aspect of the need to explore already available data in genome databases, rather than focusing efforts on sequencing more isolates.

## Author contributions

ST, EG, RK, NP, and JJ contributed to the manuscript preparation and finalizing. All authors contributed to the article and approved the submitted version.

## Conflict of interest

The authors declare that the research was conducted in the absence of any commercial or financial relationships that could be construed as a potential conflict of interest.

## Publisher's note

All claims expressed in this article are solely those of the authors and do not necessarily represent those of their affiliated organizations, or those of the publisher, the editors and the reviewers. Any product that may be evaluated in this article, or claim that may be made by its manufacturer, is not guaranteed or endorsed by the publisher.

